# A 5 item version of the Compliance Questionnaire for Rheumatology (CQR5) successfully identifies low adherence to DMARDs

**DOI:** 10.1186/1471-2474-14-286

**Published:** 2013-10-08

**Authors:** Lyndsay D Hughes, John Done, Adam Young

**Affiliations:** 1 Health Psychology Section, Institute of Psychiatry, King’s College London, 5th Floor Bermondsey Wing, Guy’s Hospital, London SE1 9RT, England; 2 Centre for Lifespan and Chronic Illness Research (CLiCIR), University of Hertfordshire, Health Research Building, College Lane, Hatfield AL10 9AB, England; 3 Department of Rheumatology, St Alban’s City Hospital, Waverley Road, St Albans, Hertfordshire AL3 5PN, England

**Keywords:** Rheumatoid arthritis, DMARDs, Medication adherence, Compliance, Questionnaire, Factor analysis

## Abstract

**Background:**

Taking DMARDs as prescribed is an essential part of self-management for patients with Rheumatoid Arthritis. To date, the Compliance Questionnaire for Rheumatology (CQR) is the only self-report adherence measure created specifically for and validated in rheumatic diseases. However, the factor structure of the CQR has not been reported and it can be considered lengthy at 19 items. The aim of this study was to test the factor structure of the CQR and reduce the number of items whilst retaining robust explanation of non-adherence to DMARDs. Such a reduction would increase the clinical utility of the scale, to identify patients with sub-optimal adherence to DMARDs in the clinic as well as for research purposes.

**Methods:**

An exploratory factor analysis was performed to reduce the number of items in the CQR and then a confirmatory factor analysis was run to establish the fit of a 5 item version (CQR5) to the data. A discriminant function analysis was performed to determine the optimal combination of questions to identify suboptimal adherence.

**Results:**

The factor analyses identified a unidimensional 5 item model that explains 50.3% of the variance in adherence and has good internal consistency and fit to the data. Discriminant function analysis shows that the CQR5 can affectively detect 69% of low adherers to DMARDs using Fisher’s weighted regression equation.

**Conclusion:**

A shortened version of the CQR increases the clinical utility by reducing the patient burden whilst maintaining a good level of reliability and validity for a short, self-administered, self-report questionnaire.

## Background

As Rheumatoid Arthritis (RA) is currently incurable, treatment is focused upon prevention of joint damage and loss of function with typical therapies including corticosteroids to reduce inflammation and DMARDs to prevent joint damage [1]. Since DMARDs have been shown to reduce disease activity and joint damage [2,3] it is therefore essential to ensure that DMARDs are being taken regularly and correctly in accordance with the clinicians’ prescription to enhance efficacy for maintaining joint function. However, it is well established that DMARDs can take months to exhibit noticeable therapeutic benefits and can sometimes have unpleasant side-effects that prompt patients to stop taking them [4].

The extent to which a patient takes medication as prescribed is termed “adherence” [5]. A patient who is fully adherent never (or rarely) misses or changes doses of the prescribed medication. Suboptimal adherence can lead to disease progression [6], increasing the burden on the healthcare system due to GP and specialist appointments and hospitalisations as well as increased morbidity and mortality [7]. The apparent treatment “failure” caused by non-adherence can lead to unnecessary treatment escalation resulting in increased costs and decreased quality of life [6-8]. Medication non-adherence in various chronic illnesses is typically quoted at 30-50% [9,10]; however, non-adherence rates for DMARDs in RA are often higher at 41% [11] and up to 75% for correct dosing [2,12].

The “gold standard” for measuring adherence is currently electronic medication event monitoring (eMEMs). Medications are decanted into specialised pots with lids equipped with microchips that automatically record the time and date it was opened, thus inferring medication taking. This allows for more accurate data regarding the timing of doses than traditional methods such as pill counts. However, eMEMs is expensive and requires numerous resources from both the patient and researcher for it to be implemented effectively. In addition, it is not suitable for extended periods which can result in “white coat compliance” where patients are deliberately more adherent for a short period because they are aware that their medication taking behaviour is being measured [12]. This method is also unsuitable for polypharmacy, limiting the ecological validity of medication taking in RA where many patients are prescribed more than one DMARD, which in itself creates challenges to adherent behaviour. To increase the utility in large scale clinical studies, the most common methods of assessing medication adherence are self-report questionnaires. Questionnaires can measure attitudes, intentions and behaviours and although they are prone to biased results from socially desirable answering, if item construction and validation is carried out correctly, these problems can be overcome. An additional advantage of questionnaires is that they can help to establish *how* and *why* a patient is non-adherent which can then be addressed, whereas eMEMs gives only the number of doses missed. Garber, Nau, Erickson, Aikens & Lawrence [13] reviewed concordance between self-reported adherence and more objective measures such as pill counts or eMEMs and found that 55% of the questionnaires were highly concordant with the objective measure. Of the self-report measures that have been developed to monitor medication adherence, most have limited sensitivity and have not been specifically developed for rheumatic diseases, which present unique barriers and procedures for treatment [14]. For this reason, the Compliance Questionnaire for Rheumatology (CQR19) was developed by de Klerk *et al.*[12,15]. This questionnaire has been validated against eMEMs [12] and has been shown to have good reliability and validity; however it can be considered lengthy for use in a clinical setting and the factor structure of the questionnaire has not been published which is necessary to aid reliability and interpretation.

Our aim therefore was to investigate whether it is possible to reduce the number of items in the CQR19 whilst retaining the internal reliability. The rationale for shortening the questionnaire was two-fold. Firstly, a condensed questionnaire would be quicker and easier to administer during a routine outpatient clinic appointment for strictly clinical uses and secondly, the reduction allows for the CQR to be incorporated more easily into a battery of questionnaires for research purposes.

## Methods

### Participants

Patients aged 18–80 years with a confirmed diagnosis of Rheumatoid Arthritis and prescribed at least 1 DMARD were opportunistically recruited from across rheumatology outpatient clinics in the UK. Patients who were not responsible for their own medication taking (i.e. relied on a carer) or could not consent for themselves were not eligible to participate.

### The Compliance Questionnaire for Rheumatology (CQR19)

The CQR19 (Table 1) is a 19 item, self-administered questionnaire that was developed with the aim of correctly identifying patients that were classified as “low” adherers (taking <80% of their medication correctly) [12,15]. The questions were identified through focus groups and clinician’s expert opinion of the likely barriers to medication taking. The four point Likert answering scale ranges from; “Definitely don’t agree” (scored 1) to “Definitely agree” (scored 4) with lower scores indicating lower levels of adherence. The CQR19 was validated against eMEMs and found to correctly identify 62% of low adherers without the extensive time and costs that are associated with “gold standard” medication monitoring techniques such as pill counting or blood chemistry levels. An additional advantage to the questionnaire is that the answers can provide some indication of the social or cognitive reasons behind non-adherence. When used in conjunction with specialised psychosocial measures, this provides the potential for healthcare professionals to address problems identified by the questionnaire as barriers to taking medication.

**Table 1:** The full compliance questionnaire for rheumatology (CQR19)

	**Questions**
**Q1**	If the rheumatologist tells me to take the medicines, I do so
Q2*	I take my anti-rheumatic medicines because I then have fewer problems
Q3*	I definitely don’t dare to miss my anti-rheumatic medications
**Q4**	If I can help myself with alternative therapies, I prefer that to what my rheumatologist prescribes
Q5*	My medicines are always stored in the same place and that’s why I don’t forget them
Q6*	I take my medicines because I have complete confidence in my rheumatologist
**Q7**	The most important reason to take my anti-rheumatic medicines is that I can still do what I want to do
**Q8**	I don’t like to take medicine. If I can do without them, I will
**Q9**	When I am on vacation, it sometimes happens that I don’t take my medicines
**Q10**	I take my anti-rheumatic drugs, for otherwise what’s the point of consulting a rheumatologist?
**Q11**	I don’t expect miracles from my anti-rheumatic medicines
**Q12**	If you can’t stand the medicines you might say: “throw it away, no matter what”
**Q13**	If I don’t take my anti-rheumatic medicines regularly, the inflammation returns
**Q14**	If I don’t take my anti-rheumatic medicines, my body warns me
**Q15**	My health goes above everything else and if I have to take medicines to keep well, I will
**Q16**	I use a dose organizer for my medications
Q17*	What the doctor tells me, I hang on to
**Q18**	If I don’t take my anti-rheumatic medicines, I have more complaints
**Q19**	It happens every now and them, I go out for the weekend and then I don’t take my medicines

Note: Items denoted with * have been retained in the final 5 item CQR5 questionnaire.

### Methodology

All consecutive patients at 3 rheumatology clinics in the UK were approached and asked if they would consent to participate in a questionnaire study. Those that gave informed consent were provided with a booklet of questionnaires, of which the CQR19 was one. Factor analysis was then carried out on the responses in order to reduce the number of items in the CQR19. Ethical approval for this study has been given by the Hertfordshire Research Ethics Committee (REC) of the UK NHS.

### Statistical analyses

#### Exploratory factor analysis (EFA)

An exploratory factor analysis (EFA) was carried out on the full CQR19 using SPSS v15 to identify the factor structure and to aid reduction of the number of items without losing reliability. The number of factors, the amount of variance explained by each factor and the amount of variance explained overall were each recorded in order to compare successive models to ensure that the explanatory ability of the questionnaire in a reduced form was not compromised. Items were selected for removal if they did not add explanatory power to the scale. One item was removed at a time, the EFA procedure repeated, and each of the criteria above inspected again to look for improvements in the reliability statistics. This procedure was continued until the internal reliability could no longer be improved by removing items.

The internal consistency measured by Cronbach’s α and percentage of variance explained of the final reduced model were evaluated against acceptable thresholds to ensure the model was stable before a confirmatory factor analysis was carried out.

#### Confirmatory factor analysis (CFA)

As the data were categorical, new polychoric and asymptotic covariance matrices were created from which the subsequent analyses were performed as there was a non-normal response pattern. Datascreening was then carried out in order to check for univariate and multivariate normality and missing cases.

In contrast to the EFA where the factor structure is dictated by the data, a confirmatory factor analysis (CFA) tests whether or not the data fits a *predefined* factor structure. Therefore, the CFA was testing whether the reduced questionnaire developed by the EFA provided a good model fit to measure medication adherence. The CFA was carried out using Lisrel 8. The reduced questionnaire was tested using Robust Maximum Likelihood and the fit to the data was evaluated using goodness of fit indices. These were chosen to include at least one index from each fit class; absolute, parsimony and comparative [16]. Taking into account the different but important ways in which these fit indices evaluate the model, including at least one of each gives the opportunity to fully consider the suitability of the model in question.

Model modification was carried out using modification indices provided by Lisrel and removing items with standardized correlated residuals higher than ±2.00 and R^2^ < 0.3 values indicating large error and little explanation of medication adherence respectively [17]. For each modification, the fit indices were again examined to determine whether further modification was required.

The items that were retained in the final model were checked to ensure that they explained more variance than by chance. In this case, each item should have a *positive* parameter estimate with a t value >1.96 (significant to α = 0.05). The final model was tested in a random sample of 500 created using the bootstrap test in Stata with the “cfa” command [18].

#### Discriminant function analysis of CQR5

In order to test the discriminant ability of the CQR5, each patient was classified as either a “high” or “low” adherer, based on the regression model given by de Klerk *et al*. [12]. A discriminant function analysis was then carried out using SPSS v15. This determined whether the model can reliably distinguish between high and low adherers. This test also gives the weights for each question to create a regression equation to be used with the reduced questionnaire to optimise classification.

## Results

### Participants

A total of 225 patients completed the CQR19. A total sample size of 225 gives a-cases-per-predictor ratio for the exploratory factor analysis of 12:1 which is in keeping with general rules of thumb suggested for this type of analysis [19]. The median age range was 50–59 years and 75.1% were female (see Table 2). Methotrexate was the most commonly prescribed DMARD (54%).

**Table 2:** Demographics of participating patients

	**N**	**Percentage**
**Total sample size**	225	
**Gender**		
Male	52	23.1
Female	169	75.1
Missing	4	1.8
**Age category**		
18–29	6	2.6
30–39	20	8.8
40–49	35	15.6
50–59	50	22.2
60–69	66	29.3
70+	44	19.6
Missing	4	1.8
**Disease duration**		
<1 year	1	0.4
1–4 years	46	20.4
5–9 years	77	34.2
10–19 years	53	23.5
20–29 years	16	7.1
30+ years	19	8.4
Missing	12	5.3

### Exploratory factor analysis of CQR19

**Figure 1:**
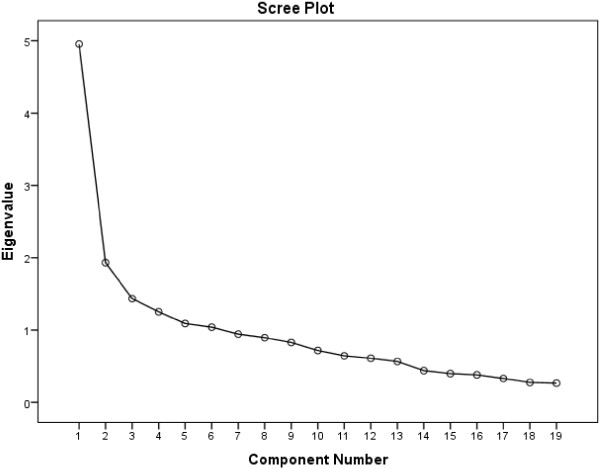
Scree plot of the CQR19.

The initial EFA of the CQR19 showed 6 eigen-values >1 which accounted for 61.6% of the variance in adherence, which is somewhat higher than the original authors found at 46% [12]. The Kaiser-Meyer-Olkin Measure of Sampling Adequacy (KMO) = 0.79 which indicates a weak factor structure [20]. This can also be seen as the eigen-values of two of the factors were very low at 1.09 and 1.04, a trait which is enhanced by the fact that the factor matrix indicated that only one item loaded highly on each of factors 2–6 suggesting that the additional factors were the product of items that inadequately measure medication adherence (see Additional file 1: Table S1). A single factor is also suggested by the Scree Plot in Figure 1.

The reduction of items led to a gradual increase in KMO and reduction in the number of factors, suggesting that the presence of more than one factor was an artefact of a few questions that in this case do not appear to be measuring the construct of medication adherence. This is supported by the fact that the percentage of variance in medication adherence explained by the questionnaire reduces by only 11% (from 61.6% to 50.3%) on removal of 8 items, and subsequently 4 factors. The results of the factor analyses are summarised in Table 3.

**Table 3:** Results of exploratory factor analyses when removing items

**Factor analysis**	**Item removed**	**MSA of removed item**	**Kaiser-Meyer-Olkin**	**Number of factors**	**Highest eigen value**	**Ratio of first to second eigen values**	**Cumulative percentage of variance explained**
CQR19	-	-	0.79	6	4.48	2.86	61.61
CQR18	11	0.39	0.80	6	4.96	2.57	64.00
CQR17	16	0.52	0.81	5	4.92	2.55	60.87
CQR16	19	0.67	0.83	4	4.79	2.97	54.92
CQR15	12	0.69	0.83	4	4.71	3.16	56.20
CQR14	9	0.68	0.84	3	4.65	3.39	51.05
CQR13	8	0.66	0.85	3	4.60	3.37	53.69
CQR12	1	0.82	0.84	2	4.30	3.36	54.97
CQR11	4	0.86	0.84	1	4.26	-	50.36

The final model that was identified from the EFA was the CQR11 which had only one factor. This showed both a good KMO (0.84) and Cronbach’s α (0.84), indicating a strong factor structure and reliable measure. This model explained 50.3% of the overall variance in medication adherence, and has the benefit of being short and robust. The items retained were; 2, 3, 5, 6, 7, 10, 13, 14, 15, 17, 18.

### Confirmatory factor analysis of CQR11

Datascreening of the polychoric and asymptotic correlation matrices showed that there were 11 missing values and 1 missing case. Once listwise deletion had been implemented, the resulting effective sample size was N = 187 giving a cases per predictor ratio of 18:1. Of these 187 cases, there were 154 distinct response patterns, indicating that the majority of participants responded completely differently to everybody else. The two most common patterns were; i) answering *“strongly agree”* to every question (N = 17) and ii) answering *“agree”* to every question (N = 7). A similar pattern was not found with the *“disagree”* responses; therefore it is possible that these participants were showing social response bias as *“agree”* responses indicate high adherence rates. However, the bivariate normality appeared to hold with nearly all of the p values being non-significant and two being very close to non-significant (item 10 *vs* item 2, p = 0.01 and item 18 *vs* item 3, p = 0.03).

The CQR11 model that was identified from the Exploratory Factor Analysis showed a less than satisfactory fit as the Satorra-Bentler χ^2^ and Root Mean Square Error (RMSEA) were outside of the acceptable ranges (see Table 4 and footnote for recommended cut off values). Model modification was therefore carried out using modification indices provided by Lisrel and removal of items with residuals of more than ±2.00 or where R^2^ < 0.3, to improve the model fit. Through subsequent modifications, it was found that 6 items consistently had the highest standardised residuals and the lowest R^2^ values, indicating that these items produced the most error and explained little of the variance in medication adherence. For these reasons, they were removed from the model to produce the CQR5 (see Table 1 for items retained in the CQR5).

**Table 4:** Confirmatory factor analysis goodness of fit statistics

**Model**	**χ**^**2**^	**Satorra-Bentler χ**^**2**^	**RMSEA (90% CI)**	**NFI**	**CFI**	**RMR**
CQR11	465.34**	172.05**	0.11 (0.095;0.13)	0.94	0.95	0.094
CQR5	56.64**	14.14*	0.089 (0.036;0.15)	0.98	0.99	0.054

** = p < 0.001.

* = p < 0.05.

For the purposes of this analysis, the following fit indices were used with the respective cut off values as recommended by Hu and Bentler [21]. Absolute fit class; χ^2^ and Satorra-Bentler Scaled χ^2^ because the latter adjusts for polychoric correlation (p > .05), Root Mean square Residual (RMR; <.08). Parsimony fit class; Root Mean Square Error of Approximation (RMSEA; <.08). Comparative fit class; Normed Fit Index (NFI; >.9) and Comparative Fit Index (CFI; >.9).

**Figure 2:**
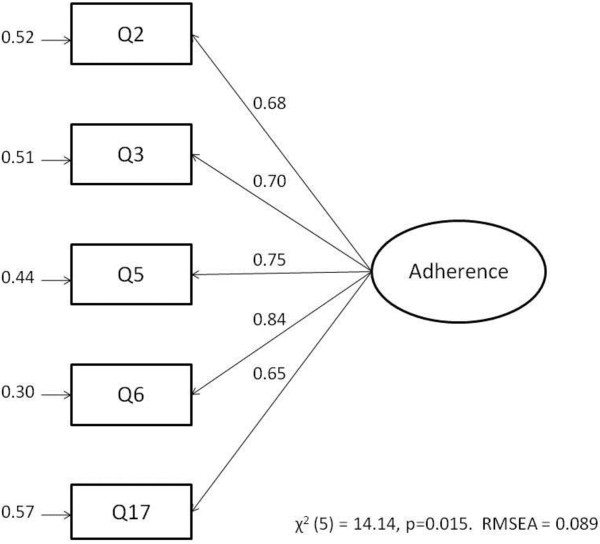
Path diagram for CQR5.

The fit indices for the CQR5 were much improved on the CQR11 and showed an acceptable model. The fit indices were all within acceptable parameters; RMSEA = 0.089 (95% CI, 0.036:0.15), NFI = 0.98, CFI = 0.99 and RMR = 0.054 indicating a good fit to the data. Although the χ^2^ values were beyond those acceptable for a good fit, these are susceptible to sample sizes >100 making p > 0.05 overly sensitive and so should be interpreted in conjunction with other indices [21]. All of the t-values for the parameter estimates were positive and highly significant, which is indicated by the large parameter estimates shown in Figure 2. The R^2^ values for the parameter estimates ranged from R^2^ = 0.65 (Q17) to R^2^ = 0.84 (Q6) showing that each of the items explained a large amount of variance in medication adherence. The internal reliability was acceptable as Cronbach’s α = 0.85. The goodness of fit indices remained adequate in a bootstrapped sample of 500 randomly selected samples from the dataset.

### Discriminant function analysis

A discriminant function analysis was carried out using the CQR5 to identify the ideal weighted combination of items to identify high and low adherers measured by the full CQR19. The canonical linear discriminant analysis was highly significant, F(5, 228) = 45.10, p < 0.001, indicating that the CQR5 can effectively discriminate between high and low adherers. The effect size η^2^ was large at 0.50 and the explained variance was very high at 70.5%. The structure matrix indicates that Q3 (0.84), Q17 (0.30) and Q5 (0.20) were most strongly correlated with the function of adherence. This is supported by a one way ANOVA of the item means between groups which shows that these questions produce significantly lower means by patients classified as low adherers, indicating that they “do not agree” more often than those classified as high adherers.

The CQR5 correctly predicted group membership measured by the full CQR19 for 88.5% of cases, with sensitivity to predict low adherence of 69% and specificity of predicting high adherence of 97%.

The structure matrix gives the optimal linear combination of the CQR5 questions to maximise the discriminant ability. Fisher’s classification function coefficients result in the following equations:

(1) $$\begin{array}{ll} \;D_{0}=- & 27.611+\left(4.407*Q2\right)+\left(0.939*Q3\right)\\ & +\left(6.101*Q5\right)+\left(2.366*Q6\right)+\left(2.531*Q17\right) \end{array}$$

(2) $$\begin{array}{ll} \;D_{1}=- & 33.304+\left(2.801*Q2\right)+\left(5.008*Q3\right)\\ & +\left(6.471*Q5\right)+\left(1.215*Q6\right)+\left(3.252*Q17\right) \end{array}$$

Given the two parameters D_0_ and D_1_, if D_0_ is greater than D_1_ then the respondent should be classified as likely to be a low adherer. Conversely, if D_1_ is greater than D_0_ then the respondent should be classified as likely to be highly adherent. See Additional file 2.

## Discussion

The present study found that reducing the CQR to just 5 items did not dramatically reduce its explanatory power and the sensitivity of identifying low adherers remained high. The exploratory factor analysis allowed for the factor structure to be made simpler and more robust with the removal of extraneous items. This reduces the burden on patients whilst completing the questionnaire, and makes interpretation easier as all items can be reliably considered to be related to adherence.

The confirmatory factor analysis confirms that the CQR5 fits the data well and explains 52.9% of variance in medication adherence which is good for a very short, self-administered questionnaire. The CQR5 also performed at a similar rate in a bootstrapped sample of 500 repetitions, confirming that the scale had not been over-fitted ad hoc to this particular dataset, but that it is likely to be applicable to the wider RA population.

Further evidence of this generalisability is shown by the fact that two of the items identified in the CQR5 (Q3 and Q5) correspond to two of the four items that the original authors found to explain 35% of the variance in their sample [12]. As the full CQR19 only explained 46% of the variance in their sample, these items are clearly contributing highly to the explanation of medication adherence.

It is interesting to note that all six of the reversed items were removed from the questionnaire by the EFA. This suggests that there was a fundamental problem with the delivery of these questions that led to them not explaining non-adherence in an adequate way. Four of these six items explore issues of medications in general and the patients’ expectations of their medications. These constructs are well measured in Horne *et al.*[22] Belief’s about Medications Questionnaire (BMQ) which has been tested and found to be reliable and valid across a number of illnesses including RA [23]. It could therefore be suggested that the BMQ should be used in conjunction with the CQR to specifically address this construct. The two other reversed items are concerned with reduced medication adherence on weekends and holidays. Unsurprisingly, it was found that these two items were strongly correlated, although neither of them correlated strongly with any other item. It was interesting to find that Q9; “When I am on vacation, it sometimes happens that I don’t take my medications” showed no correlation to Q14; “If I don’t take my anti-rheumatic medication regularly, the inflammation returns”. This could be due to the fact that either patients had not experienced taking a holiday whilst on their current DMARD, or that patients view their holidays as completely separate to their normal lives and develop a new routine that is unconcerned with their disease status. This possibly changing belief would be interesting to investigate in a more formal way, particularly if it leads to low levels of adherence whilst away from patients’ normal care teams.

The discriminant function analysis of the CQR5 suggests good specificity by identifying 97% of the high adherers classified by the full CQR19 and sensitivity of 69% at identifying low adherers. As the CQR5 is a nested model of the CQR19, which was used as the dependent variable to classify patients, good sensitivity and specificity would be expected. However, the good discriminatory power that the CQR5 shows provides more evidence that the other 14 items of the CQR19 are extraneous and are not providing additional explanatory power over and above the five retained items, demonstrating the clinical utility of the more parsimonious questionnaire. The CQR5 performs equally as well as the CQR19 at correctly classifying patients, but with only a quarter of the number of questions. However, as the CQR19 was used as the dependent variable for the discriminant function analysis, the sensitivity and specificity of the CQR5 should be interpreted with caution unless it is shown to be as good when using an objective measure of adherence (such as electronic medication event monitoring) as the dependent variable.

As was found by de Klerk *et al.*[12], the CQR5 is most predictive when used as a weighted discriminant equation. This is the optimum combination of weighted questions to classify patients as either high or low adherers, and is the function that should be used when implementing the CQR5. The structure matrix indicates that Q3; “I definitely don’t dare to miss my anti-rheumatic medication” is the most indicative of high or low adherence as high adherers tend to “agree” whereas low adherers tend to “disagree”. It may therefore be possible to get an indication of the overall result from the answer to this question with a positive (answer 3 or 4) response indicating high adherence and a negative (answer 1 or 2) response indicating low adherence. The main benefit of using self report questionnaires is to attempt to identify the determinants of non-adherence in order to target for intervention. Although the CQR is not designed for this purpose, it does allow for the latent variable of adherence to be measured alongside validated measures of adherence predictors. Questionnaires such as the revised Illness Perception Questionnaire (IPQ-R) [24] and the Beliefs about Medications Questionnaire (BMQ) [22] have shown promising strides in identifying determinants of non-adherence in many illnesses including Rheumatoid Arthritis [23-26]. The CQR5 can be used in future research as a short, parsimonious, unidimensional adherence scale to successfully identify the most useful areas for intervention with the ultimate aim of improving patient outcomes.

This study benefitted from having a large sample size and patients that had a wide range of disease and treatment experience as well as differing socio and geographic demographics and ages. Although the CQR5 performed well compared to the CQR19, the next step would be to validate the reduced version against a measure of adherence such as eMEMs to determine the sensitivity of identifying suboptimal adherence in a more objective manner. However, some support is given by the fact that within this sample of 225 patients, 24% (N = 53) were classified as being low adherers based on their CQR5 scores which is in accordance with previously published figures [5,12,23,27] of medication non-adherence.

## Conclusions

This study shows that it is possible to reduce the number of questions of the CQR19 without losing its explanatory properties, thus improving the clinical utility. The CQR5 is as good as the CQR19 at classifying patients as low adherers and is quick and easy enough to be widely used in the clinic without burdening patients. The simplified factor structure is also more parsimonious and gives an indication to the clinical care team of possible sub-optimal DMARD adherence which can then be addressed. This then has the potential to improve the prognosis for the patient and avoid unnecessary treatment escalations or delays.

## Competing interests

The authors declare that they have no competing interests.

## Authors’ contributions

LH designed the study, collected the data, carried out the analysis and wrote the draft of the manuscript. JD participated in the design of the study, provided assistance with data analysis and contributed to the draft of the manuscript. AY contributed to the design of the study, collected data and contributed to the draft of the manuscript. All authors have read and approved the final manuscript.

## Pre-publication history

The pre-publication history for this paper can be accessed here:

http://www.biomedcentral.com/1471-2474/14/286/prepub

## Supplementary Material

Additional file 1: Table S1 Factor Matrix for CQR19 Exploratory Factor Analysis. Click here for file

Additional file 2 CQR5 Adherence calculator. Click here for file
