# Supplementary material for: A 5 item version of the Compliance Questionnaire for Rheumatology (CQR5) successfully identifies low adherence to DMARDs
Source: BMC Musculoskelet Disord. 2013 Oct 8;14:286. doi: 10.1186/1471-2474-14-286 (PMC3852995; doi:10.1186/1471-2474-14-286)
Supplement: Additional file 1: Table S1 — Factor Matrix for CQR19 Exploratory Factor Analysis. [file 1471-2474-14-286-S1.docx]

Table 1: Factor Matrix for CQR19 Exploratory Factor Analysis

| **Factor Matrix^a^** | | | | | | |
| --- | --- | --- | --- | --- | --- | --- |
|  | Factor | | | | | |
|  | 1 | 2 | 3 | 4 | 5 | 6 |
| CQR1 | .541 | -.055 | -.306 | -.007 | -.181 | .121 |
| CQR2 | .719 | -.155 | .107 | -.123 | -.322 | .195 |
| CQR3 | .610 | .136 | -.013 | .123 | -.006 | .111 |
| CQR4r | .236 | .120 | .031 | -.213 | .165 | .054 |
| CQR5 | .528 | .087 | -.295 | -.007 | -.048 | -.074 |
| CQR6 | .608 | -.073 | -.372 | -.113 | -.062 | .115 |
| CQR7 | .456 | -.225 | -.008 | -.274 | -.105 | .005 |
| CQR8r | .255 | .145 | .011 | -.025 | .058 | .254 |
| CQR9r | .336 | .615 | .020 | -.020 | .057 | .023 |
| CQR10 | .330 | -.274 | -.103 | .168 | .022 | -.108 |
| CQR11r | -.015 | -.025 | .224 | .174 | -.083 | .162 |
| CQr12r | .312 | .313 | .168 | -.454 | .265 | .048 |
| CQR13 | .638 | -.283 | .392 | .051 | -.023 | -.008 |
| CQR14 | .579 | -.306 | .320 | .095 | .008 | -.119 |
| CQR15 | .550 | .082 | -.145 | -.073 | .122 | -.268 |
| CQR16 | .186 | .008 | -.021 | .304 | .316 | .407 |
| CQR17 | .578 | -.104 | -.316 | .245 | .248 | -.159 |
| CQR18 | .632 | -.160 | .320 | .018 | .191 | -.110 |
| CQR19r | .436 | .807 | .180 | .253 | -.231 | -.142 |
| Extraction Method: Unweighted Least Squares. | | | | | | |
| a. 6 factors extracted. 20 iterations required. | | | | | | |

Table 2: Factor Matrix for CQR18 Exploratory Factor Analysis

| **Factor Matrix^a^** | | | | | | |
| --- | --- | --- | --- | --- | --- | --- |
|  | Factor | | | | | |
|  | 1 | 2 | 3 | 4 | 5 | 6 |
| CQR1 | .538 | -.055 | -.279 | .120 | -.190 | .049 |
| CQR2 | .703 | -.139 | .124 | .146 | -.256 | -.061 |
| CQR3 | .608 | .139 | -.045 | -.072 | -.042 | .116 |
| CQR4r | .236 | .122 | .071 | .172 | .179 | .081 |
| CQR5 | .525 | .080 | -.245 | .054 | -.086 | -.066 |
| CQR6 | .611 | -.076 | -.320 | .249 | -.125 | .081 |
| CQR7 | .466 | -.235 | .052 | .324 | -.036 | -.257 |
| CQR8r | .261 | .163 | .039 | .115 | -.088 | .411 |
| CQR9r | .334 | .612 | .008 | .009 | .039 | .009 |
| CQR10 | .329 | -.272 | -.131 | -.136 | .014 | -.026 |
| CQr12r | .316 | .334 | .252 | .394 | .378 | .006 |
| CQR13 | .644 | -.281 | .408 | -.151 | -.068 | .073 |
| CQR14 | .579 | -.298 | .305 | -.198 | -.001 | .032 |
| CQR15 | .541 | .074 | -.100 | -.003 | .142 | -.149 |
| CQR16 | .177 | .009 | -.070 | -.110 | .077 | .318 |
| CQR17 | .609 | -.129 | -.462 | -.285 | .369 | -.035 |
| CQR18 | .629 | -.150 | .306 | -.116 | .169 | -.032 |
| CQR19r | .434 | .807 | .094 | -.298 | -.201 | -.165 |
| Extraction Method: Unweighted Least Squares. | | | | | | |
| a. 6 factors extracted. 15 iterations required. | | | | | | |

Table 3: Factor Matrix for CQR17 Exploratory Factor Analysis

| **Factor Matrix^a^** | | | | | |
| --- | --- | --- | --- | --- | --- |
|  | Factor | | | | |
|  | 1 | 2 | 3 | 4 | 5 |
| CQR1 | .541 | -.054 | -.317 | .063 | -.180 |
| CQR2 | .716 | -.146 | .087 | .093 | -.354 |
| CQR3 | .603 | .137 | -.042 | -.073 | -.044 |
| CQR4r | .236 | .128 | .075 | .208 | .103 |
| CQR5 | .526 | .085 | -.268 | .001 | -.024 |
| CQR6 | .609 | -.070 | -.356 | .187 | -.105 |
| CQR7 | .459 | -.213 | .010 | .252 | -.078 |
| CQR8r | .247 | .144 | .024 | .083 | -.106 |
| CQR9r | .334 | .623 | .017 | -.013 | .016 |
| CQR10 | .331 | -.279 | -.136 | -.142 | .115 |
| CQr12r | .322 | .357 | .273 | .505 | .226 |
| CQR13 | .644 | -.286 | .396 | -.131 | -.072 |
| CQR14 | .584 | -.307 | .306 | -.177 | .045 |
| CQR15 | .544 | .081 | -.101 | .002 | .194 |
| CQR17 | .581 | -.111 | -.352 | -.158 | .387 |
| CQR18 | .629 | -.153 | .323 | -.057 | .179 |
| CQR19r | .429 | .770 | .089 | -.357 | -.111 |
| Extraction Method: Unweighted Least Squares. | | | | | |
| a. 5 factors extracted. 15 iterations required. | | | | | |

Table 4: Factor Matrix for CQR16 Exploratory Factor Analysis

| **Factor Matrix^a^** | | | | |
| --- | --- | --- | --- | --- |
|  | Factor | | | |
|  | 1 | 2 | 3 | 4 |
| CQR1 | .550 | .166 | -.265 | -.208 |
| CQR2 | .728 | -.110 | .061 | -.383 |
| CQR3 | .582 | .151 | .034 | .017 |
| CQR4r | .231 | .175 | .210 | .046 |
| CQR5 | .516 | .231 | -.176 | -.008 |
| CQR6 | .619 | .225 | -.241 | -.163 |
| CQR7 | .477 | -.065 | .007 | -.173 |
| CQR8r | .235 | .158 | .131 | -.092 |
| CQR9r | .259 | .437 | .248 | .085 |
| CQR10 | .355 | -.194 | -.278 | .118 |
| CQr12r | .288 | .324 | .477 | .064 |
| CQR13 | .660 | -.413 | .219 | -.014 |
| CQR14 | .605 | -.416 | .112 | .095 |
| CQR15 | .539 | .173 | -.016 | .204 |
| CQR17 | .590 | .097 | -.356 | .379 |
| CQR18 | .637 | -.263 | .225 | .187 |
| Extraction Method: Unweighted Least Squares. | | | | |
| a. 4 factors extracted. 9 iterations required. | | | | |

Table 5: Factor Matrix for CQR15 Exploratory Factor Analysis

| **Factor Matrix^a^** | | | | |
| --- | --- | --- | --- | --- |
|  | Factor | | | |
|  | 1 | 2 | 3 | 4 |
| CQR1 | .552 | .245 | -.064 | -.233 |
| CQR2 | .725 | -.115 | .178 | -.304 |
| CQR3 | .581 | .138 | .140 | .105 |
| CQR4r | .213 | .077 | .154 | .091 |
| CQR5 | .517 | .283 | -.025 | -.034 |
| CQR6 | .624 | .301 | -.050 | -.228 |
| CQR7 | .477 | -.074 | -.019 | -.244 |
| CQR8r | .228 | .104 | .241 | -.003 |
| CQR9r | .245 | .353 | .402 | .268 |
| CQR10 | .369 | -.071 | -.303 | -.008 |
| CQR13 | .670 | -.466 | .135 | .066 |
| CQR14 | .610 | -.420 | -.028 | .109 |
| CQR15 | .532 | .166 | -.023 | .189 |
| CQR17 | .607 | .236 | -.439 | .272 |
| CQR18 | .624 | -.310 | .014 | .171 |
| Extraction Method: Unweighted Least Squares. | | | | |
| a. 4 factors extracted. 5 iterations required. | | | | |

Table 6: Factor Matrix for CQR14 Exploratory Factor Analysis

| **Factor Matrix^a^** | | | |
| --- | --- | --- | --- |
|  | Factor | | |
|  | 1 | 2 | 3 |
| CQR1 | .550 | .282 | .172 |
| CQR2 | .720 | -.118 | .303 |
| CQR3 | .568 | .092 | .046 |
| CQR4r | .201 | .012 | .036 |
| CQR5 | .509 | .275 | .062 |
| CQR6 | .623 | .330 | .196 |
| CQR7 | .479 | -.036 | .154 |
| CQR8r | .217 | .031 | .179 |
| CQR10 | .373 | .020 | -.175 |
| CQR13 | .677 | -.471 | -.002 |
| CQR14 | .620 | -.388 | -.128 |
| CQR15 | .520 | .142 | -.107 |
| CQR17 | .621 | .358 | -.507 |
| CQR18 | .627 | -.327 | -.154 |
| Extraction Method: Unweighted Least Squares. | | | |
| a. 3 factors extracted. 18 iterations required. | | | |

Table 7: Factor Matrix for CQR13 Exploratory Factor Analysis

| **Factor Matrix^a^** | | | |
| --- | --- | --- | --- |
|  | Factor | | |
|  | 1 | 2 | 3 |
| CQR1 | .548 | .304 | -.138 |
| CQR2 | .770 | -.118 | -.520 |
| CQR3 | .561 | .104 | -.003 |
| CQR4r | .196 | .014 | -.001 |
| CQR5 | .507 | .303 | .039 |
| CQR6 | .609 | .344 | -.063 |
| CQR7 | .486 | -.012 | -.168 |
| CQR10 | .372 | .019 | .175 |
| CQR13 | .675 | -.447 | .034 |
| CQR14 | .622 | -.388 | .158 |
| CQR15 | .522 | .160 | .171 |
| CQR17 | .587 | .278 | .329 |
| CQR18 | .629 | -.324 | .174 |
| Extraction Method: Unweighted Least Squares. | | | |
| a. Attempted to extract 3 factors. More than 25 iterations required. (Convergence=.001). Extraction was terminated. | | | |

Table 8: Factor Matrix for CQR12 Exploratory Factor Analysis

| **Factor Matrix^a^** | | | |
| --- | --- | --- | --- |
|  | Factor | | |
|  | 1 | 2 | 3 |
| CQR2 | .718 | -.128 | -.364 |
| CQR3 | .556 | .126 | -.015 |
| CQR4r | .196 | .023 | -.017 |
| CQR5 | .498 | .333 | -.080 |
| CQR6 | .580 | .323 | -.160 |
| CQR7 | .500 | .012 | -.292 |
| CQR10 | .370 | .042 | .167 |
| CQR13 | .700 | -.429 | .044 |
| CQR14 | .648 | -.362 | .196 |
| CQR15 | .523 | .217 | .084 |
| CQR17 | .592 | .372 | .352 |
| CQR18 | .654 | -.238 | .123 |
| Extraction Method: Unweighted Least Squares. | | | |
| a. 3 factors extracted. 5 iterations required. | | | |

Table 9: Factor Matrix for CQR11 Exploratory Factor Analysis

| **Factor Matrix^a^** | | |
| --- | --- | --- |
|  | Factor | |
|  | 1 | 2 |
| CQR2 | .688 | -.074 |
| CQR3 | .557 | .134 |
| CQR5 | .509 | .368 |
| CQR6 | .580 | .345 |
| CQR7 | .489 | .040 |
| CQR10 | .369 | .025 |
| CQR13 | .712 | -.440 |
| CQR14 | .647 | -.355 |
| CQR15 | .525 | .212 |
| CQR17 | .561 | .270 |
| CQR18 | .659 | -.242 |
| Extraction Method: Unweighted Least Squares. | | |
| a. 2 factors extracted. 5 iterations required. | | |
